# Physicians' Pocket Visiting List, Diary and Almanac for 1853

**Published:** 1852-10

**Authors:** 


*Physician's Pocket Visiting List, Diary and Almanac for* 1853. Philadelphia, Lindsay & Blakiston.

This is a very useful Diary and note book for a physician. We are particularly pleased to see in it a memorandum of poisons and their antido tes. Every physician should carry one of these little books in his pocket—generally a vacant place in a doctor's garment. It contains a visiting list for thirty patients for every day in the year; obstetric and vaccination engagements, and for accounts asked for and delivered. A book like this is needed by dentists.

